# Supplementary material for: Development of Novel Bioluminescent Biosensors Monitoring the Conformation and Activity of the Merlin Tumour Suppressor
Source: Int J Mol Sci. 2024 Jan 26;25(3):1527. doi: 10.3390/ijms25031527 (PMC10855677; doi:10.3390/ijms25031527)
Supplement: Supplementary file 1 [file ijms-25-01527-s001.zip › ijms-2819435-Table S1.pdf]

Table S1. List of Primers Used for PCR

Construct	Primer Name	Sequence (5’ to 3’)
WT Mer-Intra-BS	EcoR1-Merlin-NoStart-F	actgggaattcagccggggccatcgcttcccg
	Nhe1-STOP-SmBiT-Linker-(NoStop)Merlin-R	cgagctagcttacagaatctcctcgaacagccggtagccggtcacaccgctcgag cctccacctccgctcccgccaccaccgagctcttcaaagaaggcc
A585W Mutant	NF2-A585W-mut-F	agctcaccttgcagagcTGGaagtcccgagtggccttc
	NF2-A585W-mut-R	gaaggccactcgggacttCCAgctctgcaaggtgagct
S518A mutant	NF2-S518A-mut-F	actgacatgaagcggcttGCCatggagatagagaaagaa
	NF2-S518A-mut-R	ttctttctctatctccatGGCaagccgcttcatgtcagt
S518D mutant	NF2-S518D-mut-F	actgacatgaagcggcttGACatggagatagagaaagaa
	NF2-S518D-mut-R	ttctttctctatctccatGTCaagccgcttcatgtcagt
ΔEL mutant	EcoR1-Merlin-NoStart-F	actgggaattcagccggggccatcgcttcccg
	NheI-Stop-SmBiT-Merlin-593R	GCAGCTAGCTTA CAG AAT CTC CTC GAA CAG CCG GTA GCC GGT CAC ttcaaagaaggccactcgggac
